# HIP Surgical Techniques to Enhance Rehabilitation (HIPSTER): a single-centre, double-blind, parallel three-arm, randomized-controlled, superiority trial

**DOI:** 10.1302/2633-1462.68.BJO-2025-0065

**Published:** 2025-08-27

**Authors:** Holly Whitmore, Alison Smeatham, Siobhan Creanor, Fiona C. Warren, Sarah L. Whitehouse, Elizabeth Gordon, Timothy P. Holsgrove, A. M. Kassam, HIPSTER Research Team

**Affiliations:** 1 Research and Development, Princess Elizabeth Orthopaedic Centre, Royal Devon and Exeter University Healthcare NHS Foundation Trust, Exeter, UK; 2 Exeter Clinical Trials Unit, University of Exeter, Exeter, UK; 3 Hip Unit, Princess Elizabeth Orthopaedic Centre, Royal Devon and Exeter University Healthcare NHS Foundation Trust, Exeter, UK; 4 Queensland University of Technology, Brisbane, Australia; 5 Research and Development, Royal Devon and Exeter University Healthcare NHS Foundation Trust, Exeter, UK; 6 Department of Engineering, Faculty of Environment, Science and Economy, University of Exeter, Exeter, UK; 1 Royal Devon University Healthcare NHS Foundation Trust (RDUH), Exeter, UK; 2 University of Exeter, Exeter, UK

**Keywords:** Total hip replacement, Hip arthroplasty, HIPSTER, RCT, Clinical trial, SPAIRE, rehabilitation, hips, patient-reported outcome measures (PROMs), tendons, arthroplasty, posterior approaches, piriformis, Total hip arthroplasty (THA), randomized-controlled trial, Body mass index

## Abstract

**Aims:**

The primary aim of this trial is to investigate whether two novel robotic-assisted tendon-sparing posterior approaches to total hip arthroplasty (THA) surgery, the piriformis-sparing posterior approach (PSPA) and the spare piriformis and internus, repair externus technique (SPAIRE), improve early patient outcomes in THA compared with a robotic-assisted standard posterior approach (PA).

**Methods:**

HIP Surgical Techniques to Enhance Rehabilitation (HIPSTER) is a single-centre, double-blind, parallel three-arm, individually randomized, controlled, superiority trial. A total of 309 participants aged over 18 years who have been listed for an elective THA will be recruited. Participants will be randomized in a 1:1:1 ratio to SPAIRE:PSPA:PA, using minimization (with a random element) on sex (males; females), age (< 50 years; ≥ 50 years), and BMI (< 30 kg/m^2^; ≥ 30 kg/m^2^). The primary outcome is the patient-reported outcome of Oxford Arthroplasty Early Recovery Score (OARS), assessed six weeks after surgery. Secondary outcome measures include blood biomarkers, activity monitoring, and patient-reported outcome measures.

**Conclusion:**

The trial will assess whether the two novel robotic-assisted tendon-sparing posterior approaches to THA surgery, the PSPA and SPAIRE, improve patient outcomes in THA compared with a robotic-assisted standard PA. If successful, it is anticipated that the results of this trial will provide the evidence necessary to plan a future multicentre, randomized-controlled trial to compare the best-performing tendon-sparing approach (PSPA or SPAIRE) identified in this efficacy trial with the gold standard PA, to assess whether the efficacy results are generalizable across the NHS. At the time of the submission, the trial is currently completing recruitment, and the follow-up will be completed in 2026.

Cite this article: *Bone Jt Open* 2025;6(8):991–1005.

## Introduction

Musculoskeletal disorders are the leading global cause of years lived with disability.^1^ Osteoarthritis (OA) is a key contributor to this, and 8% of the UK population aged over 45 years (2.1 million people) have sought treatment for OA of the hip.^2^ Total hip arthroplasty (THA) surgery can be used to replace both the ball (femoral head) and socket (acetabular) parts of the hip with artificial components to restore the joint, with the aim of providing relief from pain and improving mobility.

THA is a highly successful surgical procedure, with over 100,000 THAs performed annually in the UK. The National Joint Registry of England, Wales, Northern Ireland, and the Isle of Man (NJR) and the Swedish Hip Register (SHR) report similar ten-year survivorship of primary THA implants of approximately 95%.^3,4^ However, patient-reported outcome measures (PROMs) show that 12 months (52 weeks) after THA surgery, a substantial proportion of patients (12.3%) report moderate to severe pain in the operated hip, 7.4% of patients have severe problems or are unable to complete their usual daily activities, and 6.3% are dissatisfied or very dissatisfied with their operation.^4^ Furthermore, all these patient outcomes worsen by the time of five-year and ten-year follow-up.^4^ These results demonstrate that, while clinical outcomes such as survivorship of the implant are extremely good for THA, there remain significant challenges to overcome in terms of post-surgical pain, activity, and patient satisfaction.

There are several surgical approaches that can be used for THA, but the posterior approach (PA) is used in 54% to 66% of primary THA procedures in the UK, Sweden, New Zealand, and the USA,^4-7^ and is estimated to be the most commonly used approach worldwide.^6,8-11^

**Fig. 1:**
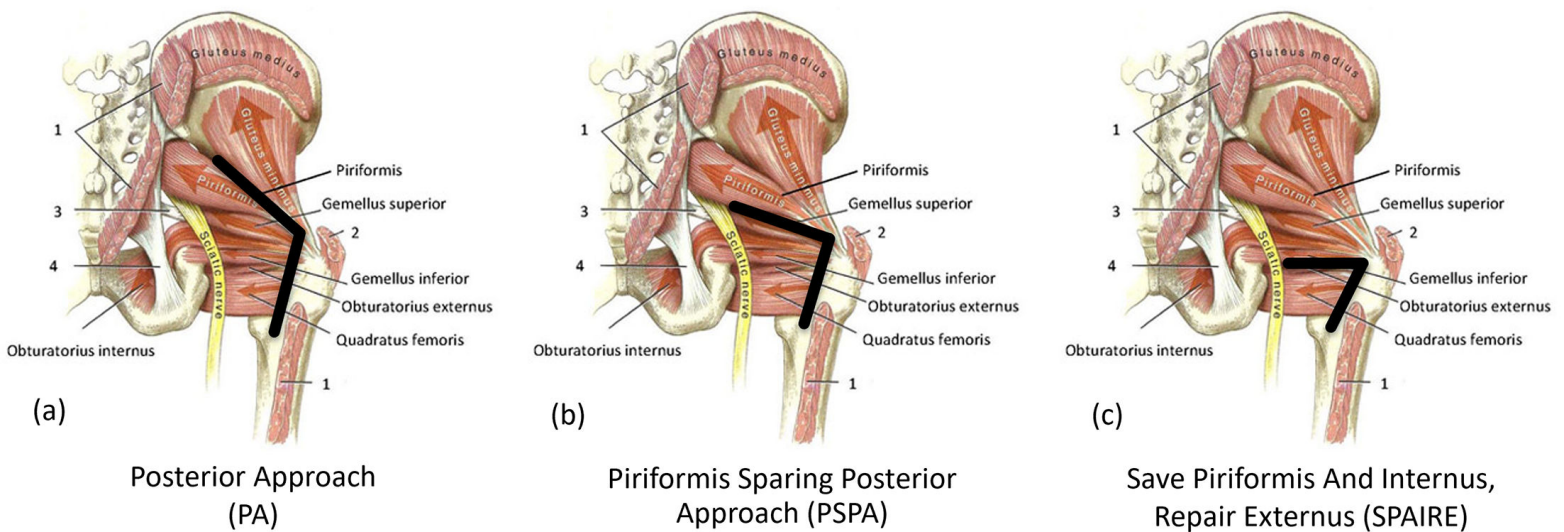
The three surgical versions of the posterior approach (PA) that will be investigated in the trial, with the lines of dissection and tendon release denoted with thick black lines. PA releases three tendons (a), the piriformis sparing posterior approach (PSPA) releases two (b), and the spare piriformis and internus, repair externus technique (SPAIRE) approach releases only one tendon (c). (Adapted with permission from A. Neumann, Kinesiology of the Musculoskeletal System – Foundations for Rehabilitation, second edition, Chapter 12 Hip, p. 496, Elsevier (2010)).

The PA (Figure 1a) involves cutting (releasing) and then repairing three musculotendinous structures (piriformis, obturator internus, and obturator externus). Releasing and repairing tendons during THA has been shown to lead to increased fatty infiltration in the muscle, and a reduction in muscle volume,^11-13^ which may have a detrimental effect on muscle function. Three months after THA using a PA, the operated side is significantly weaker than the contralateral side in terms of hip external rotation (50%) and hip extension (26%),^14^ and this weak external rotation is still present 12 months after surgery.^15^ Tendons that are released and subsequently repaired have an increased risk of failure, with failure of tendon repair reported to be as high as 86% (43/50) in patients following arthroplasty.^16^ This evidence suggests that releasing and repairing tendons during THA using a PA has the potential for increased pain and reduced function following THA, and these factors may be responsible for the cohort of patients who remain dissatisfied even after a technically well implanted THA.

Given these challenges, modified PAs have been developed to reduce musculo-tendinous damage during surgery to improve postoperative stability, proprioception, and rehabilitation. These modifications include the piriformis sparing posterior approach (PSPA) and spare piriformis and internus, repair externus technique (SPAIRE)^17^ (Figure 1).

The increased challenge of component positioning in tendon-sparing THA approaches such as the PSPA and SPAIRE techniques can be negated with the use of robotic guidance. Domb et al^18^ reported that the use of the MAKO robotic arm-assisted surgery system (Stryker, USA) results in a significantly higher proportion of acetabular components being correctly positioned in the target safe zone (100%) compared with conventional THA (80%) using the PA. A follow-up retrospective review trial of 1,980 THA patients further demonstrated this, with a significantly higher proportion of acetabular components positioned in the target safe zone using a robotic-assisted PA (98%) compared with non-assisted PA (70%).^19^ Robotic-guided surgery is increasingly being used nationally and internationally, and provides the opportunity to utilize more soft-tissue preserving surgical approaches. While the above modified PAs have been proposed and used clinically, to date there is no high-quality evidence to evaluate whether they have an impact on patient outcomes post-surgery. Therefore, a trial is needed to assess the efficacy of tendon-sparing posterior approaches in robotic-assisted THA.

## Objectives

The aim of this single-centre, double-blind, parallel three-arm, randomized-controlled, superiority trial is to investigate whether two novel robotic-assisted tendon-sparing posterior approaches to THA surgery, the PSPA and SPAIRE, improve patient outcomes in THA compared with the robotic-assisted standard PA.

### Primary objective

The primary objective is to determine whether there is a clinically significant between-group difference comparing PSPA and PA, and SPAIRE and PA, for THA patients using the Oxford Arthroplasty Early Recovery Score (OARS)^20^ patient-reported outcome measure (PROM) at six weeks postoperatively, and to estimate the magnitude of any between-group differences (Table I and Table II). The OARS is a 14-question PROM that has been designed to assess early recovery following THA. It covers four principal domains: pain; sleep; nausea/feeling unwell; and mobility.

**Table I.** Estimands table for the primary research question.

Target population	Anyone with OA of the hip requiring a total hip arthroplasty. The trial will focus on patients suitable for a cementless acetabular component as this is appropriate for robotic guided surgery.
Variable of interest	The primary outcome measure is the OARS PROM at 6 weeks postoperatively
Key intercurrent events	Death: while alive policy: data collected up to point of death Treatment switching: participant receives a hip arthroplasty via surgical approach that is not the one they were allocated to (due to error or surgical decision at point of treatment): - Treatment policy: data analyzed as though intercurrent event did not occur - Principal stratum: data analyzed for the known sub-population of participants who received their procedure as allocated Failure of treatment initiation: participant does not receive hip arthroplasty at all (participant characteristics, service delivery issues): - Treatment policy: cannot be applied to OARS, as only applicable postoperatively - Principal stratum: data analyzed for the known subpopulation of participants who received their procedure as allocated (assumed that failure of receipt of THA would apply regardless of allocated treatment)
Population-level summary of variable	Between-group mean difference for OARS at 6 weeks postoperatively for: (i) PSPA vs PA; (ii) SPAIRE vs PA

OA, osteoarthritis; OARS, Oxford Arthroplasty Early Recovery Score; PA, posterior approach; PROM, patient-reported outcome measure; PSPA, piriformis-sparing posterior approach; SPAIRE, spare piriformis and internus, repair externus technique; THA, total hip arthroplasty.

**Table II.** Primary and secondary objectives and outcomes.

Objective	Outcome measures	Time point(s) of evaluation of this outcome measure
**Primary objective**		
To test whether the SPAIRE and PSPA provide benefits to participants compared to the PA	OARS	Six weeks postoperatively
**Secondary objectives**		
To compare differences between PSPA and PA, and between SPAIRE and PA in terms of participant muscle damage and global inflammation	Blood biomarkers CK and CRP	At preoperative assessment (baseline), and at day 0/1 and six weeks postoperatively
To compare differences between PSPA and PA, and between SPAIRE and PA in terms of walking and sleep	Activity monitoring based on two weeks’ continuous data collection: - Mean daily steps - Mean step rate - Mean walking bout time - Maximum walking bout time - Mean daily sleep time - Mean daily sleep quality	At preoperative assessment (baseline), and at six weeks, six months (26 weeks), and 12 months (52 weeks) postoperatively
To compare differences between PSPA and PA, and between SPAIRE and PA in terms of early change	OACS	Six weeks postoperatively
To compare differences between PSPA and PA, and between SPAIRE and PA in terms of satisfaction	SAPS	At six weeks, six months (26 weeks), and 12 months (52 weeks) postoperatively
To compare differences between PSPA and PA, and between SPAIRE and PA in terms of PROMs	Pain, function, activity and mobility, and general health status and quality of life: - OHS - LEFS - EQ-5D-5L	At preoperative assessment (baseline), and at six weeks, six months (26 weeks), and 12 months (52 weeks) postoperatively
To compare the trajectory of outcome measures between SPAIRE, PSPA, and PA, and evaluate whether there are differences in functional outcomes over time	Activity monitoring based on two weeks’ continuous data collection: - Mean daily steps - Mean step rate - Mean walking bout time - Maximum walking bout time - Mean daily sleep time - Mean daily sleep quality Pain, function, activity, general health status, and satisfaction: - OHS - LEFS - EQ-5D-5L - SAPS	At six weeks, six months (26 weeks), and 12 months (52 weeks) postoperatively
To compare differences between PSPA and PA, and between SPAIRE and PA in terms of clinical outcomes during and immediately following surgery	Duration of surgery Blood loss Length of hospital stay	Intra-operatively (duration of surgery; blood loss) Until time of hospital discharge (length of hospital stay)
To compare differences between PSPA and PA, and SPAIRE and PA in terms of pain medication used in addition to the standardised prescribed post-surgery medication, immediately post-surgery and six weeks postoperatively	Additional over-the-counter or prescribed analgesia needed during the first six weeks postoperative? (Yes/No and list any additional medication)	At six weeks postoperatively
To evaluate the safety of PSPA and SPAIRE approaches with respect to the PA	Adverse events recorded from the day of surgery	Until 12 months (52 weeks) postoperatively via medical notes and at six weeks, six months (26 weeks), and 12 months (52 weeks) postoperatively

CK, creatine kinase; CRP, C-reactive protein; EQ-5D-5L, EuroQol five-dimension five-level questionnaire; LEFS, lower extremity functional scale; OACS, Oxford Arthroplasty Early Change Score; OARS, Oxford Arthroplasty Early Recovery Score; OHS, Oxford Hip Score; PA, posterior approach; PROM, patient-reported outcome measure; PSPA, piriformis-sparing posterior approach; SAPS, self-administered patient satisfaction scale; SPAIRE, save piriformis and internus repair externus.

### Secondary objectives

The secondary objectives are to compare differences between SPAIRE, PSPA, and PA in terms of; muscle damage and global inflammation using blood biomarkers; length of hospital stay, duration of surgery, blood loss; participant walking (daily steps, step rate, and walking bout time), and sleep (daily sleep time and sleep quality) parameters measured via an activity monitor; PROMs; additional analgesic use from immediately post-surgery up to six weeks postoperatively. We will also use the postoperative follow-up data to compare the trajectory of outcome measures between the three groups and evaluate whether there are any differences in outcomes over time, investigate potential mechanisms of action of the three forms of posterior approach in terms of their effect on OARS, using mediation analysis with creatine kinase (CK) and C-reactive protein (CRP) as mediators of OARS, and evaluate safety of PSPA and SPAIRE, by reporting related adverse events and serious adverse events. Primary and secondary objectives and outcomes are outlined in Table II, and the schedule of events including primary and secondary outcome measurement timepoints is shown in Table III.

**Table III.** Schedule of events.

Assessment/visit	Preoperative				Postoperative				
	Screening	Preop assessment (baseline)	7 days prior to surgery (research team)	24 hrs prior to surgery (surgeon and theatre staff)	Day 0/1 (inpatient)	Notes review after discharge	6 wks (± 2 wks)	6 mths (26 wks) (-4 wks/ +12 wks)	12 mths (52 wks) (-4 wks/ +12 wks)
Check eligibility and provide PIS	X								
Confirm inclusion/exclusion criteria		X							
Consent		X							
Demographic data (name, DOB, sex of patient, height, weight, BMI, ethnicity), any existing comorbidities, and contact details		X							
Blood biomarkers – CK and CRP		X			X		X		
Activity monitor issued in clinic and prepaid return envelope provided		X					X		
PROMs – OHS, LEFS, EQ-5D-5L in clinic		X							
Randomization			X	X					
Check preoperative imaging has been requested by surgeon (imaging to be done within 3 months of surgery date)		X							
Perioperative data: length of stay*, surgery time, blood loss, conversion of allocated randomization approach†						X			
PROMs – OARS, OACS, OHS, LEFS, EQ-5D-5L, SAPS in clinic							X		
Activity monitor issued via post and prepaid returns envelope provided								X	X
PROMs – OHS, LEFS, EQ-5D-5L, SAPS remotely								X	X
Additional analgesia use							X		
Safety reporting – AEs and SAEs					X	X	X	X	X
Additional surgical procedures								X	X

* Length of stay is recorded at time of discharge, i.e. number of nights a participant stays in hospital post-surgery.

† Conversion of approach records if the intended approach is converted to another during surgery (SPAIRE to PSPA or PA; or PSPA to PA).

AE, adverse event; BMI, Body mass index; CK, creatine kinase; CRP, C-reactive protein; DOB, date of birth; EQ-5D-5L, EuroQol five-dimension five-level questionnaire; LEFS, lower extremity functional scale; OACS, Oxford Arthroplasty Early Change Score; OARS, Oxford Arthroplasty Early Recovery Score; OHS, Oxford Hip Score; PIS, patient information sheet; PROMs, patient-reported outcome measures; SAE, severe adverse event; SAPS, self-administered patient satisfaction scale.

### Trial design and setting

The trial is a single-centre, double-blind, parallel, three-arm, individually randomized, controlled, superiority trial. The trial will run at the Royal Devon and Exeter Hospital (Wonford) (RDE), which encompasses the South West Ambulatory Orthopaedic Centre (SWAOC) at the Nightingale Hospital Exeter (NGE) surgical site, under the Royal Devon University Healthcare NHS Foundation Trust (RDUH) Eastern Services, which is a centre of excellence for hip arthroplasty. The chief investigator (CI) is Al-Amin Kassam, who will also act as the site principal investigator (PI); the co-chief investigator (co-CI) is Timothy P. Holsgrove. The trial is funded by the Efficacy and Mechanism Evaluation (EME) Programme, a Medical Research Council (MRC), and National Institute for Health and Care Research (NIHR) partnership.

## Participant identification

### Inclusion criteria

Adult patients aged 18 years or over who are listed for elective THA surgery at the RDUH (either RDE or NGE), using a cementless acetabular component are included. Patients with OA of the hip, with any BMI, and willing and able to provide informed consent in English.

### Exclusion criteria

Excluded from the trial are patients with active systemic or local infection that would preclude standard THA surgery; patients undergoing bilateral THA in same operative episode; patients unable to give informed consent; and patients who are unable or unwilling to take part in the trial process, including patients unable to undertake physical activity monitoring data collection or complete the PROMs questionnaires in English.

## Trial treatments

### Preoperative imaging and surgical planning (all participants)

As part of the routine MAKO planning procedure, participants in all three groups will have a spiral CT scan and an anteroposterior (AP) radiograph of both hips. Imaging will be undertaken according to the needs of the MAKO system and a 3D plan will be made for the surgeon for every participant. The CT for the planning procedure will not determine or affect a patient’s eligibility for involvement in the trial.

In order to produce the plan, the CT and radiograph images will be sent to Stryker. This is used for all patients undergoing MAKO THA as part of routine clinical practice; this includes all patients who are not involved in the research trial. The plan provided by Stryker will describe the optimal implant size and position for restoration of leg length, offset, hip centre of rotation, and stability. This will be provided for all participants, regardless of treatment allocation. During the operation, the surgeon may make adjustments to this according to their normal practice in any of the trial groups.

### Surgical expertise

It is a prerequisite that all treating surgeons listed on the trial delegation log have been trained to use the MAKO system, have performed a sufficient number of robotic-assisted THA procedures outside of the trial, and are familiar with all of the surgical techniques used in the trial.

Because participants in all three groups are receiving a version of the PA with or without tendon preservation, if there is a clinical need to release more tendons to perform surgery safely this can be undertaken by the operating surgeon. The most common reason for conversion is if surgical exposure is insufficient and further tendon release is required to improve visualization of the acetabulum to enable preparation and implantation of the acetabular component. This is likely to take the form of releasing one or two further tendons to improve surgical exposure. The data of any conversions will be collected; treatment switching will be accounted for using sensitivity analyses as per the estimands framework (Table I).

### Group 1: THA with the standard PA and robotic guidance (control group)

Participants will receive a THA using the PA in which three tendons are released to access the hip joint (piriformis, obturator internus, and obturator externus). These are repaired once the hip arthroplasty components have been implanted. This is the current approach used in the majority of THA procedures in the UK. Robotic guidance using the MAKO robotic system will ensure that the acetabular component is accurately positioned in the surgically planned position during surgery.

### Group 2: THA with the PSPA and robotic guidance (intervention group A)

Participants will receive a THA using the PSPA in which two tendons are released to access the hip joint (obturator internus and obturator externus). These are repaired once the hip arthroplasty components have been implanted. This is currently the most commonly used approach by surgeons in Exeter. This approach has been previously shown in a randomized controlled trial (RCT) to limit access and visibility of the hip joint compared with the PA and presents a greater challenge in accurately positioning the acetabular component.^21^ Robotic guidance using the MAKO robotic system will ensure that the acetabular component is accurately positioned during surgery.

### Group 3: THA with the SPAIRE approach and robotic guidance (intervention group B)

Participants will receive a THA using the SPAIRE approach in which only one tendon is released to access the hip joint (obturator externus). This tendon is repaired once the hip arthroplasty components have been implanted. This approach has been previously used but limits access and visibility of the hip joint, compared with both the PA and PSPA, and presents a greater challenge in accurately positioning the acetabular component. Robotic guidance using the MAKO robotic system will ensure that the acetabular component is accurately positioned during surgery.

### Post-surgical rehabilitation programme

Postoperative rehabilitation will be provided as per local NHS standard care for THA, which comprises daily physiotherapy provided postoperatively for the duration of each participant’s stay in hospital. The routine outpatient clinic follow-up appointment at six weeks postoperatively includes a review by a physiotherapist, advice on postoperative activity, and a home exercise programme. Additional outpatient physiotherapy requests are made by clinicians on case-by-case basis according to clinical need.

## Protocol procedures

### Recruitment

The trial will recruit adult patients undergoing elective THA surgery due to OA of the hip. The RDE completes approximately 800 elective, primary THA procedures each year, from which the trial participants will be recruited.

The MAKO robotic guidance system currently only supports the use of cementless hip socket components, hence all patients will be assessed for their suitability for a cementless socket component based on the quality of their bone stock, which will be determined from preoperative plain radiographs.

**Fig. 2:**
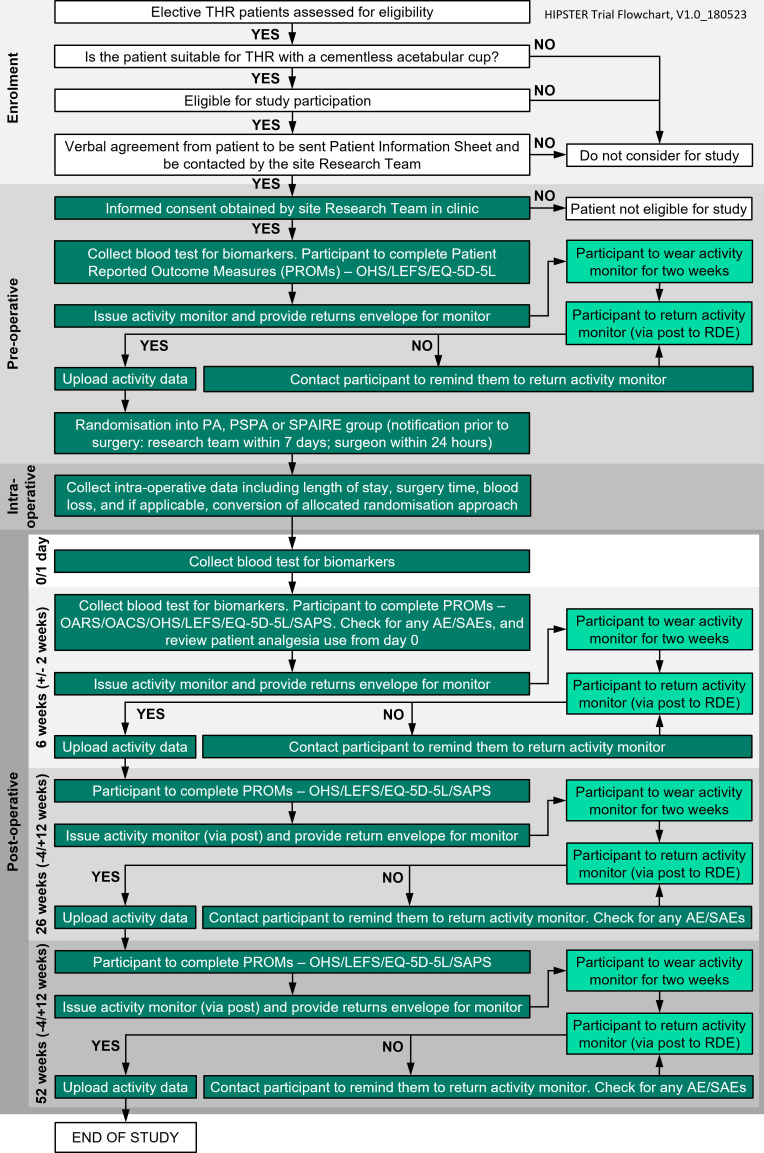
Trial pathway. AE, adverse event; EQ-5D-5L, EuroQol five-dimension five-level questionnaire; LEFS, lower extremity functional scale; OACS, Oxford Arthroplasty Early Change Score; OARS, Oxford Arthroplasty Early Recovery Score; OHS, Oxford Hip Score; PSPA, piriformis-sparing posterior approach; PA, posterior approach; SAE, serious adverse event; SAPS, self-administered patient satisfaction scale; SPAIRE, save piriformis and internus repair externus; THR, total hip replacement.

The trial’s recruitment period is 21.5 months with recruitment anticipated to be completed in August 2025. Figure 2 outlines the trial pathway for participants.

### Screening and eligibility assessment

There are two ways in which potentially eligible patients will be identified:

1) A patient listed for THA surgery using the MAKO system will appear on a waiting list review conducted by a member of the direct care team (DCT) at the RDUH. This will be repeated regularly throughout the trial to ensure maximum identification of all eligible patients. The electronic medical records of each patient will be reviewed against the trial inclusion and exclusion criteria by a delegated clinician. Patients aged under 65 years will be contacted by a member of the DCT and asked for permission for a member of the site research team to post or email a patient information sheet (PIS) to them. The records and radiographs of patients aged over 65 years will be further reviewed by a surgeon to confirm whether or not they may be suitable for a cementless socket and therefore are potential participants. Those who are likely to be suitable will then be contacted following the above process. The age of 65 years has been selected as patients aged under 65 years have been shown to potentially have lower revision rates using uncemented acetabular components in the NJR. Patients aged above 65 years can benefit from cemented or cementless acetabular components depending on the clinical records and radiographs and so the decision of which component should be used, and therefore whether MAKO robotic surgery can be used, will be determined by the treating clinical team.

2) If a patient has not yet been listed for THA surgery, the trial can be introduced to them by their clinician during their routine outpatient assessment clinic, once the clinician has reviewed and assessed the patient against the trial inclusion and exclusion criteria. The patient will be asked for permission for a member of the site research team to post or email a PIS to them and then clearly documented in the patient’s electronic medical notes on EPIC (the electronic health record system used for patients at the RDUH).

### Informed consent

Members of the site research team approved to obtain informed consent will arrange to meet with the patient at either routine surgical consenting clinic or preoperative assessment appointment. During this appointment the trial will be discussed in depth, giving the patient the opportunity to ask questions they have concerning trial participation. The patient will decide whether or not they wish to take part, and if they agree to participate they will be asked to provide their written consent using the trial informed consent form (ICF). The site research team will explain to the patient that participating in the trial is entirely voluntary and that they have the right to withdraw at any time without needing to provide any reason, or they have the option to flexibly change their participation in the trial by selectively reducing or ceasing any or all of the following aspects:

1) Use of the physical activity monitor at any timepoint, pre- and postoperatively
2) Collection of blood biomarkers at any timepoint, pre- and postoperatively
3) Completion of PROMs data at any timepoint, pre- and postoperatively
4) Passive data collection from routine medical records (except where required for reporting of serious adverse events or for monitoring purposes)

The consent discussion will be documented in the patient’s electronic medical notes on EPIC by the person who took consent, and the patient will be linked to the trial on EPIC. This ensures other clinical teams are aware of their enrolment and ensures monitoring of any serious adverse events that may occur during the trial period.

The participant’s general practitioner (GP) will be informed of their inclusion into the trial. In addition, once the trial has ended, the GP will be sent a letter notifying them of the treatment allocation received by the participant.

When a patient consents to participate in the trial, they will be asked whether they would like to be kept informed about developments of the trial. At the end of the trial, all participants will receive a letter notifying them of the treatment allocation they received.

### Randomization

Participants will be randomized in a 1:1:1 ratio to SPAIRE:PSPA:PA by the Exeter Clinical Trials Unit (ExeCTU). To promote balance across participant characteristics thought to be predictive of outcome, minimization with a random element will be used to allocate participants to the groups. Characteristics of sex (males; females), age (< 50 years; ≥ 50 years), and BMI (< 30 kg/m^2^; ≥ 30 kg/m^2^) will be used in the minimization algorithm (i.e. a total of eight combinations of minimization factors). The operating surgeon will not be included as a minimization factor due to anticipated low interoperator variability; however, a sensitivity analysis will be performed for the primary outcome measure, to investigate any operator effects.

Participants will be randomized via the REDCap database (Research Electronic Data Capture, created by Vanderbilt University, with study data collected and managed using REDCap version 13.7.31 electronic data capture tools developed and hosted by Exeter CTU, University of Exeter, UK) within the seven days prior to the planned surgery date (Table III). This is to ensure randomizations are able to be performed around weekends and public holidays and in the working hours of ExeCTU and to ensure surgeons are aware of the randomization prior to surgery on weekend operating lists or ready for first cases on the operating lists on a Monday morning. Randomizations will be performed, so far as is practically feasible, in strict order by date of scheduled surgery. The operating surgeon and allocated members of theatre staff will be notified of the randomized allocation directly from REDCap within 24 hours prior to surgery.

### Blinding

The trial will be double-blinded; the participants will be unaware of which type of surgical approach they have received, and identified members of the site research team, which will include the research nurse and research practitioners who collect outcome data, will also be blinded to the allocated group. Surgeons will remain blinded until they receive an email with the randomized allocation within 24 hours of the scheduled surgery (Table III). Operating surgeons will not be involved in collecting any postoperative data. A senior statistician will be unblinded throughout the trial to facilitate provision of unblinded reports to the data monitoring committee (DMC). Following completion of the blinding of trial statisticians (BOTS) guidance tool,^22^ the risk of having an unblinded analyzing statistician throughout the trial up to the point of performing the primary analyses was considered to be low with regard to participant safety, the trial conduct and procedures, and integrity of the trial results. Nevertheless, the trial statistician (performing data analysis) will remain blinded until the presentation of the primary analyses of the primary and secondary outcome data. At this point, the wider trial team will be unblinded. The remaining analyses will then be performed with the statistician unblinded.

We do not expect a situation to arise where knowledge of the exact surgical technique used will be needed to inform any urgent medical management. As such, no special emergency unblinding process is required.

### Withdrawal from the trial and changes to participation status

Participant withdrawal will be completed in accordance with the principles of the PeRSEVERE project (PRincipleS for handling end-of-participation EVEnts in clinical trials REsearch).^23^ Participants have the right to withdraw at any time during the trial without prejudice to their clinical care and there is no obligation to provide reasons for withdrawal. At the time of providing informed consent, participants must be made aware that they need to contact the site research team should they no longer wish to participate in the trial. For each withdrawal or change in participation status, the withdrawal log on REDCap is to be completed by a member of the site research team, which will trigger a notification to the trial manager that a participant has been withdrawn, and the reason why (if provided).

If a patient withdraws their consent prior to randomization, or if they are excluded on the grounds of ineligibility post-consent but prior to randomization, they will be fully withdrawn from the trial (i.e. no further data collection); the data collected up until the point of withdrawal will still be available for use in trial reporting. The site research team will endeavour to replace such individuals. If a participant wishes to withdraw post-randomization and prior to surgery, then the participant would either receive standard THA or be withdrawn from surgery entirely if it is no longer required. Participants who withdraw at this stage will not be replaced and follow-up data will not be collected. At the discretion of the trial CI, participants may be withdrawn from randomized surgery and/or from follow-up data collection if it is felt in their best interest to do so.

For randomized participants, all data collected up to the point of change in their participation status or withdrawal will be retained and used in the analysis. For individuals who have provided informed consent, but who later lose capacity during the trial the following process will apply; the consent obtained prior to loss of capacity will not endure the loss. The participant will stop their participation, but data already collected up to the point of loss of capacity will be retained and will be used in analysis.

### Sample collection, transportation, analysis, and storage

Obtaining blood samples will be coordinated by the site research team. All samples will be handled and processed by the local laboratories at the RDUH, adhering to all relevant Trust standard operating procedures (SOP) and working instructions.

### Co-enrolment

Any co-enrolment to other research studies will need to be approved by the CI and the site research team.

### End of trial

All participants will be actively followed up until their 12-month (52-week) follow-up (Table III), and at this timepoint, the participant will complete their involvement in the trial. The end of the trial as a whole will be after all trial participants have completed all follow-ups, i.e. when the last participant follow-up at 12 months (52 weeks) postoperatively has been completed, no further follow-up is planned, all data queries have been resolved, the database locked, the analysis completed, and the results published. At this point, participants will revert to the standard follow-up protocol of the unit, and they and their GP will be sent a notification of treatment allocation letter informing them of which THA technique the participant received.

## Safety reporting

Reportable adverse events (AEs) (Table IV) should be recorded from the point of surgery to the end of trial participation. Some events which occur during treatment and recovery will be considered normal aspects of the anaesthetic and postoperative recovery process, and that occur frequently after surgery. They will not need reporting as AEs, unless in the opinion of the CI are untoward, excessive, or outside of what might normally be expected for the procedure, or fall into the category of a serious adverse event (SAE) (Table IV). Some events will be considered reportable expected AEs (or SAEs, if they meet the criteria). In certain cases, the diagnoses will be confirmed, where there is uncertainty, by the treating clinician.

**Table IV.** General definitions.

Term	Definition
AE	Any unintentional, unfavourable clinical sign or symptom, or any new illness or disease or the deterioration of existing disease or illness.
SAE	A SAE is considered to be any event that leads to: Death Serious deterioration in the health of the participant that results in: - life-threatening illness or injury; - permanent impairment of a body structure or function; - inpatient hospitalization or prolongation of existing hospitalization; - medical or surgical intervention to prevent life-threatening illness or injury or permanent impairment to a body structure or a body function - chronic disease Other important medical events Other ‘important medical events’ may also be considered serious if they jeopardize the participant or require an intervention to prevent one of the above consequences.
RUSAE	A related and unexpected SAE is an event that is related to the intervention, and ‘unexpected’ – that is, the type of event is not listed in the protocol as an expected occurrence.

AE, adverse event; RUSAE, related and unexpected serious adverse event; SAE, serious adverse event.

Causality of reportable SAEs will be assessed by the CI (in their role as site PI, or authorized delegate). All SAEs which are possibly, probably, or definitely related to the intervention will be categorized as ‘related’. If the CI or delegate is unable to assign causality within 24 hours of the site becoming aware of the event, the SAE will be treated cautiously and subjected to expedited reporting.

### Reporting adverse events

SAEs must be reported directly onto REDCap within 24 hours of the blinded site research team becoming aware of the event. SAEs classed as possibly, probably, or definitely related and unexpected (RUSAEs) will be reported by ExeCTU to the sponsor within 24 hours of ExeCTU staff becoming aware. The sponsor is responsible for onward reporting of RUSAEs to the Research Ethics Committee (REC) within 15 days of the event being reported to ExeCTU in REDCap. However, if the event results in death the SAE will be reported to the REC within seven days of the event being reported to ExeCTU in REDCap.

The CI (in their role as site PI) or their delegate, is responsible for signing off reportable SAEs within the REDCap system. The trial steering committee (TSC) and DMC will periodically review SAE data to determine patterns and trends of events, or to identify safety issues, which would not be apparent on an individual case basis.

### Notification of deaths

All deaths occurring after randomization until the 12-month postoperative timepoint, or participant withdrawal from the study, irrespective of their relationship to the trial treatment, should be reported to ExeCTU via REDCap as a SAE within 24 hours of identifying the death. In addition, if the CI becomes aware of a participant death outside this period, which appears to be related to the study intervention, this should also be reported as an RUSAE.

## Statistical analysis

Trial participants will be recruited from patients undergoing an elective THA who fulfil the eligibility criteria. The planned sample size is 309, achievable by recruiting a total of 87 participants per treatment group while allowing for a 15% drop out rate, becoming 103 per allocated group (309 in total). A detailed statistical analysis plan (SAP) has been developed during the recruitment phase of the trial, and has been approved by the TSC (Supplementary Material).

### Primary outcome analysis

The primary outcome, OARS measured at six weeks’ follow-up, will be analyzed using linear regression modelling with adjustment for minimization factors (it is noted that OARS is not completed/assessed at baseline as it would not be appropriate to do so). For each of the two pairwise comparisons with PA (i.e. PSPA vs PA and SPAIRE vs PA), the between-group mean difference will be reported with a CI and p-value. To account for the fact that there are two comparisons of interest, the threshold for statistical significance for the primary outcome will be 0.025 (two-sided) in each pairwise comparison; this approach is consistent with the approach taken for the sample size calculation. In alignment with the significance threshold, a two-sided 97.5% CI will be reported, In addition, a global p-value comparing means across all three treatment groups will be reported, plus the mean difference for SPAIRE vs PSPA and the associated two-sided 95% CI.

Baseline descriptive data will be scrutinized for balance across the three treatment groups. Should any participant characteristics be considered unbalanced, and thought to be predictive of outcome, sensitivity analyses will be performed to adjust for these characteristics, using the methods described above. Further sensitivity analyses will account for clustering by surgeon, by using a mixed model with a random effect on surgeon, in addition to adjustment for minimization factors.

### Secondary outcome analysis

For the secondary outcomes, the threshold for statistical significance of pairwise comparisons will be (two-sided) 0.05; and a global p-value comparing effects across all three treatment arms will be reported. For the pairwise comparisons of interest, PSPA compared with PA, and SPAIRE compared with PA, between-group mean differences will be reported in addition to two-sided 95% CIs; no corrections will be made for multiple testing, but p-values will be interpreted in the light of the number of comparisons being made.

Continuous PROM outcomes (Oxford Arthroplasty Early Change Score (OACS),^20^ self-administered patient satisfaction scale (SAPS),^24^ Oxford Hip Score (OHS),^25-27^ Lower Extremity Functional Scale (LEFS),^28^ EuroQol five-dimension five-level questionnaire (EQ-5D-5L))^29^ will be analyzed using the same principles as for OARS; OHS, LEFS, and EQ-5D-5L will be collected at baseline, so baseline scores will be adjusted for. In addition to the four physical activity outcomes, the two sleep outcomes and the biomarkers, CK and CRP, will be analyzed using these principles.

Time to discharge from hospital is to be measured in terms of the number of nights spent in hospital. It is anticipated that the majority of participants will be discharged on the day of surgery, so the number of nights spent in hospital will be 0. The number of nights spent in hospital will be reported descriptively by participant group, and also analyzed using a negative binomial model, which will account for zero-inflation, with adjustment for minimization variables.

Analgesia outcomes will be modelled using appropriate methods (i.e. linear regression modelling for dose outcomes and logistic regression modelling for binary outcomes).

Deaths will be reported descriptively, by allocated group, for the full period of the trial from randomization to 12-month follow-up, and for the following time periods: post-randomization and preoperative; perioperative (within 24 hours of surgery); one day to six weeks postoperatively; and more than six weeks to 52 weeks postoperative.

Furthermore, the odds ratio for death (at any time during follow-up to 52 weeks postoperatively) comparing PSPA and PA, SPAIRE and PA, and SPAIRE and PSPA will be reported with two-sided 95% CIs. Sensitivity analyses with adjustment for unbalanced baseline characteristics, and using a mixed model with random effect on surgeon, will be performed for all continuous secondary outcomes, and for the number of nights in hospital.

### Supplementary analysis

For all continuous secondary outcomes collected at more than one post-randomization timepoint, a mixed model will be performed with a random effect on participant, including data collected at all time points, and making the assumption that all missing data is missing at random (MAR). The interaction between treatment and time points will be reported with a two-sided 95% CI and global p-value. These models will be performed for the treatment policy and principal stratum approaches.

In addition, missing outcome data for the primary outcome and continuous secondary outcomes will be imputed using multiple imputation with chained equations, on the assumption that missing data is MAR. For the treatment policy and principal stratum analyses, the primary analysis for each outcome will be repeated using observed and imputed data. No subgroup or interim analyses are planned.

## Data management

The handling and storage of all personal data collected during the trial will comply with the Data Protection Act 2018^30^ and the General Data Protection Regulation (GDPR).^31^

### Data collection tools and source document identification

The primary data sources will be the participant’s electronic medical notes and trial-specific electronic case report forms (eCRFs). Data will be collected contemporaneously through the trial on eCRFs with information extracted from source data within the participant’s clinical records. PROMs data will be recorded preoperatively, and at six weeks, six months (26 weeks), and 12 months (52 weeks) postoperatively with direct participant input onto REDCap. The exception is for paper PROMs, should the participant opt for this; the site research team will then transcribe the PROMs data onto REDCap once received from the participant. Results from blood biomarker collection preoperatively and at six weeks postoperatively will be obtained from participant medical notes on EPIC and transcribed by a member of the site research team onto REDCap. Physical activity monitoring data will be uploaded and stored locally, and transferred pseudonymously to members of the University of Exeter research team (co-CI) for processing.

Participants will be identified by a trial number allocated at the point of enrolment which will ensure anonymity of data recorded electronically. This will be the same as their randomization number; however, it is different from their participant screening number.

Sites will be required to answer data queries raised by ExeCTU within a timely manner within the trial database. A data cleaning work instruction will be provided to sites.

A separate electronic data capture (EDC) project will be used to store personal identifiable data (i.e. names, addresses, email addresses, telephone numbers) that will be separate from the research data. Personal data will be collected to facilitate the sharing of newsletters and trial results and assist with retention and follow-up activities. Access to the contact details will be restricted to individuals authorized by the CI. All EDC system users will require individual log-in credentials and authorization from an approved member of the trial management team before access is granted. The EDC system will incorporate role restriction such that individual users will only be able to access and enter or edit data as their individual permissions allow. The ExeCTU trial management team will run regular reports for missing data and remind sites at least monthly to enter data that are expected, and document any reasons for missing data.

### Data handling and record keeping

A data management plan (DMP) was completed prior to starting participant recruitment and will be updated throughout the trial as appropriate. Working instructions have been provided to the RDE site on record keeping and data entry processes, and will be updated as appropriate. Electronic systems have been validated, tested, and documented before starting recruitment.

### Surgical data and robotic-assisted surgery session files

Legal agreements are in place between Stryker (the manufacturer and distributor of the MAKO robotic arm-assisted system) and the RDUH to protect participants’ identity and personal information will be held confidentially in accordance with the GDPR. These protections will remain even if data are transferred outside the EU, such as to the USA. Stryker will hold data from previously received CT scans for the purposes of planning the surgery, and could potentially use it for linkage of with the session files and surgery data. However, there will be strict contracts in place to ensure data confidentiality is maintained. This data sharing has already been approved by RDE Governance and is already currently occurring for patients undergoing MAKO THAs as part of routine clinical practice.

### Data access

Access to data held at the participating site will be restricted to those holding a substantive or honorary contract for the RDE and who have a relevant purpose to access the data. Access will be granted to authorized representatives from the RDUH NHS Foundation Trust as the sponsor, as well as representatives from the University of Exeter (UoE) for the purposes of auditing and monitoring the trial. Participants will be asked to consent to representatives of the sponsor or the UoE accessing their data that is relevant to their participation in the trial.

Data entered into the EDC system will be accessed by authorized members of the trial team at the participating site and at ExeCTU. Access will be restricted with individual log-in credentials, and site and role restriction applied so that individuals can only access data appropriate to their location and role.

### Data shared with third parties

De-identified data that underlie the results reported in the trial will be deposited in the UoE Open Research repository and available for non-commercial use, on a controlled access basis, subject to suitable data requests and data sharing agreements. Data may be used for commercial purposes, according to the conditions above, but will need specific agreements in place prior to access being agreed.

## Trial management and oversight

RDUH is the named sponsor for this trial, and will be supported by the UKCRC fully registered, and British Orthopaedic Association-affiliated ExeCTU, and the Department of Engineering at the UoE. The sponsor has had input into the design of the trial but overall responsibility for the design lies with the CI and co-CI. The sponsor is responsible for authorizing the initial submission to the REC and Health Research Authority (HRA) and subsequent amendments, ensuring appropriate agreements and indemnity arrangements are in place, overseeing the conduct of the trial and ensuring it adheres to the relevant principles of Good Clinical Practice (GCP)^32^ and the UK Policy Framework for Health and Social Care Research,^33^ and for archiving at the end of the trial. The Sponsor is not responsible for, and has no involvement in the data analysis or interpretation, or writing manuscripts.

### Trial management group

The trial management group (TMG) will be composed of the CI and co-CI, trial co-applicants, trial statisticians, patient and public involvement and engagement (PPIE) lead with at least one lay representative, the Trial Manager, and a Sponsor representative. The TMG will write the protocol, SAP and participant-facing materials, obtain relevant approvals from an NHS REC and the HRA, and ensure the trial is conducted according to the relevant principles of GCP and the UK Policy Framework for Health and Social Care. The TMG will meet monthly for the first 18 months of the trial, then at least every three months thereafter to manage the day-to-day running of the trial, monitor safety, key performance indicators, and discuss and resolve emerging issues. Members of the TMG will analyze the data, interpret the analyses, write reports to the funder, and write and submit manuscripts to peer-reviewed journals.

### Trial steering committee

The TSC will be composed of an independent chairperson with expert knowledge in the subject area, an independent statistician, PPIE representative, and at least one other independent professional member. The CI, co-CI, and senior trial statistician will join the TSC as observers and will not be voting members. The trial manager, trial statistician, and representatives of the sponsor and the funder will be invited to attend TSC meetings, also as acting as observers, and will not be voting members. The role of the TSC is to monitor and supervise the progress of the trial. The TSC chair and/or TSC committee will review the final protocol prior to submission to REC/HRA and the independent statistician on the TSC will approve the SAP prior to final database lock. The TSC will meet prior to recruitment commencing and at least six-monthly thereafter.

### Data monitoring committee

The DMC will be composed of a minimum of three independent professional members, including a statistician. The CI and co-CI, senior trial statistician, and trial manager will be invited to attend the open sessions of DMC meetings, but will not be voting members. The senior trial statistician will be unblinded throughout the trial, and the trial statistician will remain blinded until completion of the primary analyses for the primary and secondary outcomes. Only the unblinded statistician(s) can be invited to the closed section of DMC meetings and will prepare/review unblinded sections of the DMC report. The DMC will monitor accumulating trial data, including safety, and make recommendations to the TSC whether there are any ethical or safety issues that may necessitate a modification to the protocol or early closure of the trial. The DMC will meet prior to recruitment commencing and at least six-monthly thereafter.

### Patient advisory group

An overarching patient advisory group led by the PPIE lead will review patient-facing materials prior to ethical review and will have input into any revisions to patient-facing materials throughout the trial. This group will meet once a year.

### The site research team

The site research team will be responsible for the delivery of trial activities on site. This team consists of research nurses, research practitioners, and a trials administrator. The site research team is led by the team lead and will work closely with the trial manager/research coordinator and clinicians.

## Protocol compliance

The eCRFs and EDC system has been designed to assist in adherence to the protocol by guiding trial personnel through the assessments and data collection. The EDC system has also been validated to minimize protocol deviations. The site research team will notify the trial manager in the event of a protocol deviation or suspected or actual serious breach, by reporting directly to ExeCTU through REDCap. A deviation log will be maintained in REDCap by ExeCTU and reviewed regularly by the CI, co-CI, and the sponsor. Recurrent deviations will be discussed with the TMG and TSC, as appropriate. The trial manager will work with the site research team to identify the cause of the deviations and put in place steps to mitigate them, as appropriate. Protocol compliance will be reported at the end of the trial.

### Notification of serious breaches to GCP and/or the protocol

All suspected serious breaches will be notified to the sponsor by a member of the ExeCTU trial team within 24 hours of becoming aware of the breach, in accordance with the ExeCTU SOP. The RDE site will notify ExeCTU of the suspected serious breach in the first instance by reporting directly onto REDCap. The suspected breach will be logged on the ExeCTU quality management system (QMS). The sponsor representative will decide if the event constitutes a serious breach, and if so will report to the REC within seven days of becoming aware as per the REC SOP. In the event of a serious breach, the Sponsor, ExeCTU, and the individuals involved will work together to agree and implement a Corrective and Preventative Action (CAPA) plan and follow-up on the plan at agreed intervals to ensure effective implementation.^34^

## Patient and public involvement and engagement

The Exeter Hip Unit established a PPIE group during the development of the trial proposal, which included both patients and carers. Potential outcomes measures for the trial were explored by the research team through a PPIE workshop, facilitated by the public involvement team of the South-West Research Design Service, to explore patient and carer experiences of THA. The PPIE group was introduced to the overall research objective: to understand whether tendon-sparing techniques might improve the patient experience of THA. Outcome measures, including PROMs, performance outcome measures, and functional outcome measures, were discussed to identify the most suitable primary and secondary endpoints with which to assess the efficacy of the PSPA and SPAIRE techniques compared with the standard PA.

## Ethics and regulatory considerations and approvals

Following sponsor approval, the protocol, consent form, participant information sheet, and all patient-facing materials were submitted to and have been approved by a UK NHS REC and HRA (IRAS ref: 327702), and given local site confirmation of capacity and capability from the Research and Development Deparment. The trial is also registered on the ISRCTN registry (ref: 27974201).

The trial is conducted in compliance with the protocol, the principles of the Declaration of Helsinki,^35^ and to GCP guidelines. It is also conducted in accordance with the UK Policy Framework for Health and Social Care Research the Mental Capacity Act 2005^36^ and the Data Protection Act 2018.

### Amendments

All substantial amendments and relevant non-substantial amendments will be discussed by the TMG, who meet monthly. The CI, co-CI, and sponsor will be responsible for the final decision on making an amendment to the protocol. The approval of TSC members will be sought for substantial amendments to the protocol in advance of submitting them to the REC and/or HRA, and if necessary, a meeting of the TSC will be convened to discuss the amendment. Approval will also be sought from the Funder for all substantial amendments, and the Funder representative will be notified of relevant substantial amendments in advance of submission. A full list of all substantial and non-substantial amendments will be provided as part of regular funder reports. The current protocol document is V6.0, dated 8 August 2024.

The sponsor will decide if an amendment is substantial or non-substantial following HRA guidance. All amendments will be submitted to the NHS REC that issued a favourable opinion (if appropriate) and the HRA following the appropriate HRA amendment process in place at the time of submission.

## Dissemination and publication policy

The results of this trial will be shared with academic, clinical, patient, and public audiences. Participants will receive a plain English summary of the trial results, if they have indicated this on their consent form, and this will be provided via post or email according to their preference.

The research team will disseminate the results of the trial through leading academic journals in their field, and will engage with academic and clinical communities through academic conferences and specialist hip meetings. The results will be posted on the publicly available ISRCTN registry. The clinical team in the Exeter Hip Unit of the RDUH lead multiple hip training courses worldwide, which are attended by hundreds of surgeons each year. These courses, along with the established Exeter Hip Fellowship programme, will be used to share the results of the trial, and should it be successful, will provide training opportunities relating to the surgical approaches that are shown to be most beneficial to patients. This may additionally include the production of a video of the different surgical approaches to assist with surgical training.

PPIE events and initiatives will be held throughout the project. When patients are enrolled into the trial, they are asked whether they would like to be kept informed about developments of the trial, and whether they would be interested in attending PPIE events. These patients and participants are updated via quarterly newsletters, and provided the opportunity to attend engagement events. The engagement events will also be used to provide trial updates, but will also focus on including opportunities to co-develop materials, such as infographics, which along with the newsletters will be shared with a wider audience through the Exeter Hip Unit website and social media channels. An animation of the different surgical approaches has since been developed and shared on social media, as well as a QR link displayed on the patient information sheet.

We anticipate that the results of this trial will provide the evidence necessary to plan a future multicentre RCT to compare the best-performing tendon-sparing approach (PSPA or SPAIRE) identified in this efficacy trial with the gold standard PA, to assess whether the results are generalizable across the NHS.

**Take home message**

- The HIP Surgical Techniques to Enhance Rehabilitation (HIPSTER) trial will evaluate whether two novel tendon-sparing posterior surgical approaches improve patient outcomes compared with a standard posterior surgical approach for adults undergoing a total hip arthroplasty.

- It is a single-centre, double-blind, parallel three-arm, individually randomized controlled, superiority trial.
